# Association between obstructive sleep apnea and male reproductive function: a cross-sectional study with stratified analysis

**DOI:** 10.3389/fendo.2025.1636484

**Published:** 2025-09-17

**Authors:** Wei Zhang, Xu Wu, Yuyang Zhang, Hui Gao, Guodong Liu, Hao Geng, Ci Zou, Xiansheng Zhang

**Affiliations:** ^1^ Department of Urology, The Second Affiliated Hospital of Anhui Medical University, Hefei, China; ^2^ Department of Urology, The First Affiliated Hospital of Anhui Medical University, Hefei, China; ^3^ Anhui Province Key Laboratory of Urological and Andrological Diseases Research and Medical Transformation, Anhui Medical University, Hefei, China; ^4^ Department of Obstetrics and Gynecology, Reproductive Medicine Center, the First Affiliated Hospital of Anhui Medical University, Hefei, China

**Keywords:** obstructive sleep apnea, reproductive health, semen parameters, sex hormones, testosterone

## Abstract

**Objective:**

Obstructive sleep apnea hypopnea syndrome (OSAHS) is a common sleep disorder known to affect systemic physiology. Emerging evidence suggests a potential relationship between OSA and male reproductive health, but this association remains insufficiently characterized. This study aims to explore the link between OSA, semen quality, and sex hormone profiles in reproductive-aged men.

**Method:**

A total of 108 newly diagnosed OSA patients aged 20–40 years, who underwent full-night polysomnography (PSG) at the Respiratory Sleep Center of the Second Affiliated Hospital of Anhui Medical University between 2022 and 2024, were included. The control group consisted of 84 healthy adult males undergoing fertility evaluations during the same period at the same hospital. All participants completed physical examinations, semen analysis, and sex hormone testing. OSA severity was classified based on the apnea–hypopnea index (AHI) obtained from PSG in the patient group.

**Results:**

Compared with controls, men with OSA had significantly higher body weight and BMI, as well as lower sperm concentration (90.0 vs. 129.3 ×10^6^ml), total motility (68.6% vs. 71.0%), progressive motility (63.8% vs. 66.6%), and testosterone levels (13.1 vs. 21.8 nmol/L) (all p < 0.01). Stratified analyses showed a stepwise decline in semen parameters and oxygen saturation with increasing OSA severity. In multivariable analysis, AHI was independently associated with reduced sperm concentration (β = –0.393), total motility (β = –0.640), and progressive motility (β = –0.623) (all p < 0.001).

**Conclusion:**

OSA is independently associated with impaired semen quality and lower testosterone levels in reproductive-aged men. These findings highlight the potential reproductive consequences of untreated OSA and underscore the importance of early screening and intervention.

## Introduction

In recent decades, the average sleep duration of the global population has declined significantly, largely due to increasing work demands and greater participation in diverse leisure activities (1, 2). Among various causes of sleep disruption, obstructive sleep apnea hypopnea syndrome (OSAHS) is one of the most prevalent and clinically significant. It is characterized by recurrent episodes of partial or complete upper airway collapse during sleep, leading to intermittent hypoxia (IH), sleep fragmentation (SF), and hypercapnia, which together can contribute to multi-organ and systemic dysfunction (3, 4). Obstructive sleep apnea (OSA) is the most common form of sleep-disordered breathing in adults.

OSA is now widely recognized as a global public health concern, affecting approximately 1 billion individuals aged 30–69 years worldwide (5). It is associated with a range of adverse health outcomes, including hypertension, cardiovascular diseases (e.g., myocardial infarction, stroke), arrhythmias, type 2 diabetes, metabolic syndrome, daytime somnolence, cognitive dysfunction, and mood disorders (6). The prevalence of OSA is significantly higher in men compared to women, and studies have suggested associations between OSA and obesity, hormonal dysregulation, sexual dysfunction, and impaired reproductive function (7, 8).

Accumulating epidemiologic and clinical evidence has demonstrated a strong relationship between OSA and ED. ED in OSA patients is a multifactorial condition influenced by intermittent hypoxia, endothelial dysfunction, sleep fragmentation, hormonal imbalance, and comorbid metabolic disorders (9). These factors collectively impair vascular and neurological function, leading to sexual dysfunction and reduced quality of life (10). It is important to note that ED and male infertility are distinct clinical entities; while they may share common underlying mechanisms such as testosterone deficiency or oxidative stress (11, 12), not all individuals with ED exhibit impaired spermatogenesis, and vice versa. Therefore, this study focuses specifically on the relationship between OSA and male reproductive function, with an emphasis on semen quality and hormone profiles. In parallel, male reproductive function has shown a marked decline globally, with studies reporting significant reductions in both semen quality and sperm concentration over the past 50 years (13–16). A recent meta-analysis estimates that male fertility has declined by 40–60% in this time frame (17–19). Sleep disorders—including OSA and chronic sleep deprivation—have been increasingly implicated in this trend, potentially through hormonal alterations, oxidative stress, and disrupted testicular microenvironments (20).

Randomized controlled trials and observational studies have linked OSA to reduced semen quality and impaired sexual function (7, 8), while animal studies have shown that chronic intermittent hypoxia, mimicking the pathophysiology of OSA, can lead to testicular damage and impaired spermatogenesis (19). Despite these findings, clinical evidence directly examining the association between OSA and male reproductive function remains limited, particularly in young men of reproductive age. Based on previous evidence linking sleep-disordered breathing with reproductive and endocrine dysfunction, we hypothesized that (1) men with OSA would exhibit altered semen quality and sex hormone levels compared to healthy controls, and (2) these reproductive parameters would deteriorate progressively with increasing OSA severity. Therefore, the aim of the present study was to investigate the relationship between OSA, semen quality, and sex hormone levels, and to perform a stratified analysis based on OSA severity, in order to provide clinical insight into the potential reproductive consequences of OSA.

## Materials and methods

### Subjects

This cross-sectional study included outpatients with a preliminary diagnosis of OSA who visited the Respiratory Sleep Centers of the First and Second Affiliated Hospitals of Anhui Medical University between 2023 and 2025. All participants were initially screened using the STOP-Bang questionnaire, and the diagnosis of OSA was subsequently confirmed via full-night polysomnography (PSG).

Eligible participants were male adults aged 20–40 years. The control group consisted of healthy men of similar age who presented for fertility evaluations at the Physical Examination Center or Reproductive Medicine Center of the Second Affiliated Hospital during the same period. General information was collected, including demographic characteristics (age, height, weight) and vital signs such as heart rate, respiratory rate, and blood pressure.

Individuals were excluded if they had any severe or decompensated systemic disease that could affect sleep or reproductive function, such as chronic obstructive pulmonary disease, asthma, interstitial lung disease, neuromuscular disorders, heart failure, thyroid dysfunction, diabetes mellitus, rheumatologic diseases, or psychiatric conditions. Participants were also excluded if they were taking psychotropic drugs, hormonal therapies, or other medications affecting sleep or reproductive hormones, or if they had occupational or lifestyle factors that led to chronic sleep deprivation or reversed sleep–wake cycles (e.g., shift workers, frequent night duty personnel). In addition, individuals with known andrological conditions such as varicocele, cryptorchidism, orchitis, or a history of testicular trauma or surgery were excluded.

### Semen analysis

Semen samples were collected on-site in a dedicated private room at the Andrology Laboratory of the Reproductive Center, following 2–7 days of ejaculatory abstinence, as recommended by the WHO Laboratory Manual for the Examination and Processing of Human Semen, 6th Edition (2021). All participants were instructed to provide the sample via masturbation directly into a sterile container. Samples were analyzed within 60 minutes of collection at 37°C. Semen analysis was performed using a fully automated Sperm Quality Analyzer, version V (SQA-V; Medical Electronic Systems, Israel) by two experienced laboratory technicians blinded to clinical data. Parameters assessed included: semen volume, sperm concentration, total motility (progressive + non-progressive), progressive motility (PR), and sperm morphology. Morphology was evaluated using strict criteria under phase-contrast microscopy. Based on WHO criteria, PR < 35% was defined as asthenozoospermia (21). Subjects with known confounding urogenital conditions—including varicocele, cryptorchidism, orchitis, genital tract infection, or history of testicular surgery/trauma—were excluded at enrollment.

### Sleep monitoring

Full-night PSG was used for sleep monitoring and diagnostic confirmation of OSA. All examinations were performed in accordance with the International Classification of Sleep Disorders, Third Edition (ICSD-3) guidelines (22) Nocturnal monitoring lasted for at least 7 hours, and the PSG recordings were jointly interpreted by two experienced respiratory physicians on the following day. According to standard definitions, apnea was defined as a ≥90% reduction in airflow lasting ≥10 seconds, while hypopnea was defined as a ≥50% reduction in airflow lasting ≥10 seconds accompanied by at least a 3% drop in oxygen saturation. The severity of OSA was classified based on the apnea–hypopnea index (AHI), which reflects the number of apnea and hypopnea events per hour of sleep. Specifically, OSA was categorized as mild (AHI 5–15), moderate (AHI 15–30), or severe (AHI >30), while AHI <5 was considered normal. During PSG, both the mean pulse oxygen saturation (MSpO_2_) and lowest pulse oxygen saturation (LSpO_2_) were also recorded as indicators of nocturnal hypoxemia.

### Sex hormone measurement

Early in the morning of the day following the PSG test, about 2 ml of peripheral blood is obtained and analyzed in the Laboratory using an automated hematology analyzer. The sex hormone parameters measured include: follicle stimulating hormone (FSH); luteinizing hormone (LH); estradiol (E2); progesterone (P); testosterone (T); prolactin (PRL).

### Statistical analysis

All statistical analyses were conducted using IBM SPSS Statistics for Windows, version 24.0 (IBM Corp., Armonk, NY, USA) and GraphPad Prism 7.0 (GraphPad Software, San Diego, CA, USA). The normality of continuous variables was assessed using the Shapiro–Wilk test. As most variables were not normally distributed, non-parametric methods were applied. Continuous variables are presented as medians with interquartile ranges (IQR), and categorical variables are expressed as counts and percentages. Group comparisons between the OSA and control groups were performed using the Mann–Whitney U test for continuous variables and the chi-square test for categorical variables. For comparisons among different OSA severity groups, the Kruskal–Wallis H test was used. Correlation analyses between the AHI and reproductive parameters were performed using Spearman’s rank correlation coefficient. To further assess the independent association between AHI and semen quality, multivariable linear regression analyses were conducted, adjusting for potential confounding variables including age, body weight, body mass index (BMI), smoking status, alcohol consumption, T, and E2 levels. A two-tailed p-value < 0.05 was considered statistically significant.

## Results

### Demographic characteristics

A total of 108 patients diagnosed with OSA and 84 healthy adult males were included in this study. Participants in the OSA group were identified based on full-night PSG, while controls were recruited from individuals undergoing routine health or fertility examinations. As shown in **Table 1**, the median age was 28.50 years (IQR: 25.75–31.25) in the OSA group and 28.00 years (IQR: 25.62–30.38) in the control group, with no significant difference between the two groups (p = 0.223). Similarly, height did not differ significantly between groups (p = 0.741). However, the OSA group had significantly higher body weight (75.60 [IQR 71.14–80.06] kg vs. 67.70 [IQR 61.71–73.69, p < 0.001) and BMI (25.15 kg/m² [IQR: 23.16–27.13] vs. 24.30 kg/m² [IQR: 22.20–26.40], p < 0.001) compared with controls. Regarding lifestyle factors, the proportion of current smokers was 44.4% in the OSA group and 50.0% in the control group (p = 0.444), while alcohol consumption was reported by 41.7% and 42.8% of participants in the OSA and control groups, respectively (p = 0.868). None of the differences in smoking or alcohol use reached statistical significance.

**Table 1:** Demographic characteristics of the participants.

Demographic characteristics	OSA (n=108)	Control (n=84)	P
Age (years)	28.50 (25.75–31.25)	28.00 (25.62–30.38)	0.223
Height	1.73 (1.67-1.79)	1.71 (1.68-1.75)	0.741
Weight	75.60 (71.14–80.06)	67.70 (61.71–73.69)	<0.001
BMI (kg/m^2^)	25.15 (23.16–27.13)	24.30 (22.20–26.40)	<0.001
Personal history			
Smoking, n (%)	48(44.4%)	42(50%)	0.444
Alcohol use, n (%)	45(41.7%)	36(42.8%)	0.868

Values are presented as median (IQR) or number (%). OSA, obstructive sleep apnea; BMI, body mass index.

### Comparison of semen parameters and sex hormones

Semen parameters and sex hormone levels were compared between the OSA and control groups, as presented in **Table 2**. The median semen volume was slightly higher in the OSA group (2.50 ml [IQR: 1.68–3.33]) compared with the control group (2.00 ml [IQR: 1.75–2.25]), but the difference was not statistically significant (p = 0.060). In contrast, the sperm concentration was significantly lower in the OSA group (90.00 ×10^6^ml [IQR: 43.31–136.69]) than in controls (129.25 ×10^6^ml [IQR: 103.03–155.47], p < 0.001). Similarly, both total motility (68.60% [IQR: 43.07–94.12] vs. 71.00% [IQR: 58.19–83.81], p = 0.011) and progressive motility (PR) (63.75% [IQR: 40.38–87.12] vs. 66.60% [IQR: 54.33–78.86], p = 0.007) were significantly lower in the OSA group.

**Table 2:** Comparison of semen parameters and sex hormones with two group.

Demographic characteristics	OSA (n=108)	Control (n=84)	P
Semen parameters			
Semen volume (ml)	2.50 (1.68–3.33)	2.00 (1.75–2.25)	0.060
Sperm concentration (10^6^/ml)	90.00 (43.31–136.69)	129.25 (103.03–155.47)	<0.001
Total motility (PR+NP) (%)	68.60 (43.07–94.12)	71.00 (58.19–83.81)	0.011
PR (%)	63.75 (40.38–87.12)	66.60 (54.33–78.86)	0.007
Sex hormone parameters			
LH (mIU/ml)	3.72 (2.71–4.73)	4.27 (3.05–5.49)	0.007
FSH (mIU/ml)	3.97 (3.04–4.91)	4.76 (3.27–6.25)	0.031
T (nmol/L)	13.06 (11.08–15.04)	21.84 (18.82–24.86)	<0.001
PRL (ng/ml)	9.91 (6.89–12.93)	9.18 (5.88–12.48)	0.383
E2 (pmol/L)	128.00 (89.25–166.75)	156.00 (78.88–233.12)	0.003
P (nmol/L)	0.900 (0.35–1.45)	0.67 (0.03–1.35)	0.364

Values are presented as median (IQR). OSA, obstructive sleep apnea; PR, progressive motility; NP, non-progressive; FSH, follicle stimulating hormone; LH, luteinizing hormone; T, testosterone; PRL, prolactin; E2, estradiol; P, progesterone;

Regarding sex hormone levels, T was markedly lower in the OSA group (13.06 nmol/L [IQR: 11.08–15.04]) compared with the control group (21.84 nmol/L [IQR: 18.82–24.86], p < 0.001). Although the median levels of LH and FSH were also lower in the OSA group, the differences were less pronounced (LH: 3.72 mIU/ml [IQR: 2.71–4.73] vs. 4.27 mIU/ml [IQR: 3.05–5.49], p = 0.007; FSH: 3.97 mIU/ml [IQR: 3.04–4.91] vs. 4.76 mIU/ml [IQR: 3.27–6.25], p = 0.031). The differences in PRL, E2, and P levels between the two groups were not statistically significant (all p > 0.05).

### Stratified analysis by OSA severity

Patients with OSA were stratified into mild, moderate, and severe subgroups based on their AHI. As shown in **Table 3**, there were no statistically significant differences in age, height, weight, or BMI among the three subgroups (all p > 0.05). As expected, AHI increased progressively across the groups, with median values of 11.00 (IQR: 10.00-12.00), 17.00 (IQR: 15.5-18.5), and 33.00 (IQR: 27.50–38.50) for the mild, moderate, and severe groups, respectively. Oxygen saturation parameters declined with increasing OSA severity. The MSpO_2_ and LSpO_2_ were significantly lower in the severe group (83.50% [IQR: 82.62–84.38] and 73.50% [IQR: 72.00–75.00], respectively) than in the mild group (91.00% [IQR: 89.00–93.00] and 82.00% [IQR: 79.00–85.00], p < 0.001 for both).

**Table 3:** Stratified subgroup analysis on the degree of OSA.

Demographic characteristics	Mild OSA (n=66)	Moderate OSA (n=18)	Severe OSA (n=24)	P
Age (yr)	28.00 (25.50–30.50)	28.50 (26.00–31.00)	29.00 (26.75–31.25)	0.088
Height	1.73 (1.66–1.80)	1.74 (1.69–1.79)	1.69 (1.66–1.72)	0.353
Weight	74.42 (69.95–78.89)	76.65 (74.92–78.39)	76.37 (70.34–82.40)	0.310
BMI (kg/m2)	25.10 (23.10–27.10)	25.05 (22.55–27.55)	25.35 (23.46–27.24)	0.150
PSG parameters				
AHI	11.00 (10.00–12.00)	17.00 (15.50–18.50)	33.00 (27.50–38.50)	<0.001
MSpO2	91.00 (89.00–93.00)	90.50 (87.00–94.00)	83.50 (82.62–84.38)	<0.001
LSpO2	82.00 (79.00–85.00)	80.00 (77.50–82.50)	73.50 (72.00–75.00)	<0.001
Semen parameters				
Semen volume (ml)	2.05 (1.20–2.90)	2.50 (2.25–2.75)	2.75 (2.06–3.44)	0.502
Sperm concentration (10^6^/ml)	118.15 (77.40–158.90)	91.80 (41.30–142.30)	29.50 (2.5–66.22)	<0.001
Total motility (PR+NP) (%)	74.35 (62.40–86.30)	66.55 (35.55–97.55)	23.50 (9.04–37.97)	<0.001
PR (%)	71.35 (59.25–83.45)	63.75 (34.45–93.05)	22.65 (8.17–37.12)	<0.001
Sex hormone parameters				
LH (mIU/ml)	4.01 (3.00–5.01)	3.01 (2.09–3.93)	3.34 (2.05–4.62)	0.028
FSH (mIU/ml)	4.18 (3.29–5.06)	4.24 (3.40–5.08)	3.84 (2.76–4.92)	0.300
T (nmol/L)	13.35 (12.18–14.52)	12.51 (8.41–16.61)	12.05 (9.19–14.92)	0.727
PRL (ng/ml)	9.62 (6.59–12.65)	10.50 (7.00–14.00)	11.91 (8.83–14.99)	0.673
E2 (pmol/L)	109.00 (62.00–156.00)	124.50 (85.50–163.50)	150.50 (119.50–181.50)	0.833
P (nmol/L)	0.900 (0.22–1.58)	0.93 (0.33–1.54)	1.03 (0.47–1.59)	0.627

Values are presented as median (IQR). Abbreviations: OSA, obstructive sleep apnea; BMI, body mass index; PSG, Polysomnography; AHI, apnea hypopnea index; MSPO2, mean pulse oxygen saturation; LSpO2, lowest pulse oxygen saturation; PR, progressive motility; NP, non-progressive; FSH, follicle stimulating hormone; LH, luteinizing hormone; T, testosterone; PRL, prolactin; E2, estradiol; P, progesterone.

In terms of reproductive function, sperm concentration decreased significantly with increasing OSA severity, from 118.15 ×10^6^ml (IQR: 77.40–158.90) in the mild group to 29.50 ×10^6^ml (IQR: 2.5–66.22) in the severe group (p < 0.001). Similarly, total motility and progressive motility (PR) were substantially reduced in the severe group (23.50% [IQR: 9.04–37.97] and 22.65% [IQR: 8.17–37.12], respectively) compared to the mild group (74.35% [IQR: 62.40–86.30] and 71.35% [IQR: 59.25–83.45], p < 0.001 for both). There were no statistically significant differences in sex hormone levels (LH, FSH, T, PRL, E2, or P) among the OSA subgroups (all p gt; 0.05), although testosterone showed a non-significant decreasing trend.

**Figure 1:**
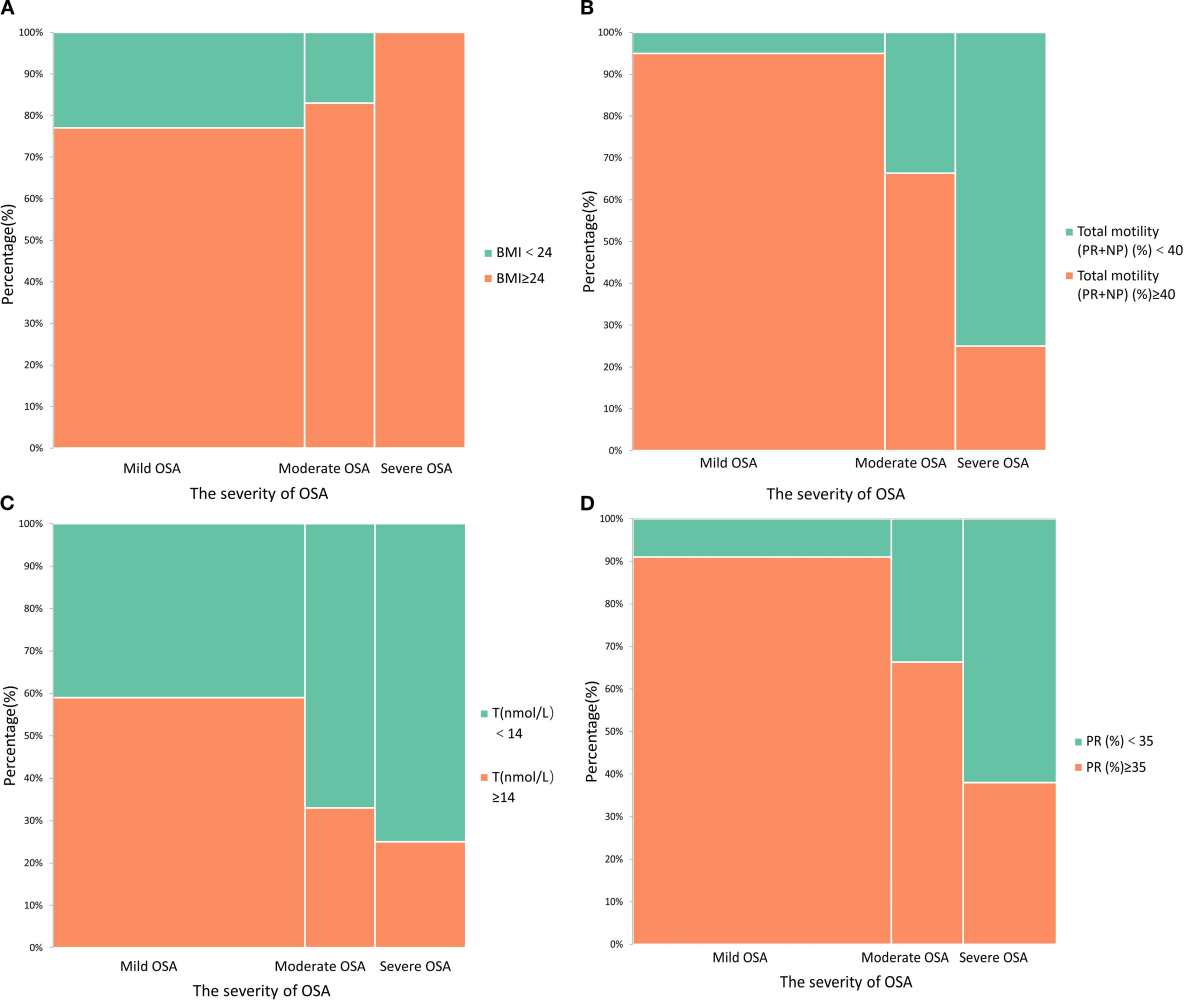
Stacked histograms demonstrate the relationship between obstructive sleep apnea (OSA) severity and other factors; **(A)** The association between obesity and OSA severity; **(B)** The effect of OSA on total sperm motility; **(C)** The association between OSA severity and testosterone deficiency; **(D)** The effect of OSA severity on progressive motility.

**Figure 1** illustrates the distribution of key reproductive and metabolic parameters across OSA severity categories. As OSA severity increased, the prevalence of obesity (BMI ≥ 24 kg/m²) rose markedly (**Figure 1A**). A similar trend was observed for reduced total motility (<40%) and progressive motility (<35%), which were more common in patients with moderate-to-severe OSA (**Figures 1B, D**). In addition, the proportion of individuals with testosterone deficiency (T < 14 nmol/L) increased with OSA severity (**Figure 1C**), further supporting the potential reproductive impact of OSA.

### Correlation between AHI and reproductive parameters

**Figure 2:**
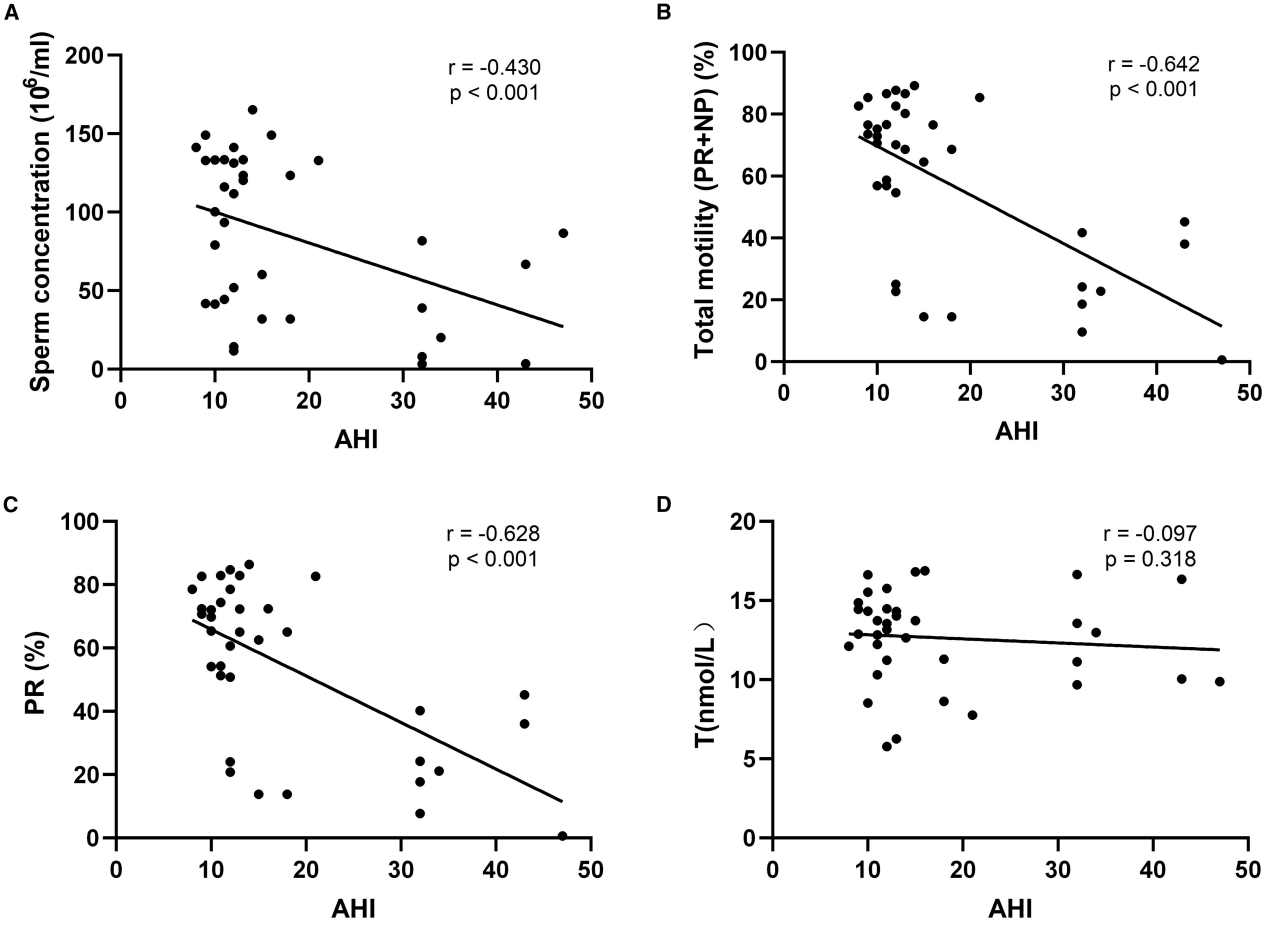
Scatterplot of the relationship between sleep apnea hypopnea index (AHI) and fertility-related indicators; **(A)** r=-0.430, P<0.001, indicating a moderate negative correlation between AHI and sperm concentration; **(B)** r=-0.642, P<0.001, indicating a strong negative correlation between AHI and total sperm motility; **(C)** r=-0.628, P<0.001, indicating a strong negative correlation between AHI and progressive motility; **(D)** r=-0.097, P=0.318, AHI had almost no correlation with testosterone level.

The associations between AHI and key reproductive parameters were evaluated using Spearman rank correlation (**Figure 2**). AHI showed significant negative correlations with sperm concentration (r = –0.430, p < 0.001), total motility (PR + NP) (r = –0.642, p < 0.001), and progressive motility (PR) (r = –0.628, p < 0.001), indicating that as OSA severity increased, semen quality declined. However, there was no statistically significant correlation between AHI and testosterone levels (r = –0.097, p = 0.318), suggesting that testosterone may not be linearly associated with OSA severity in this cohort.

### Multivariable linear regression analysis

To further explore the independent relationship between OSA severity and semen quality, multivariable linear regression analyses were performed using AHI as the predictor and semen parameters as outcomes, adjusting for potential confounders including age, body weight, BMI, smoking status, alcohol consumption, testosterone, and estradiol levels (**Table 4**).

**Table 4:** Multivariate linear regression analyses of the association between AHI and semen parameters in the OSA group.

Semen parameters	B	SE	β	t	p-value
Semen volume	0.009	0.010	0.091	0.929	0.355
Sperm concentration	-1.806	0.386	-0.393	-4.676	<0.001
Total motility (PR+NP)	-1.569	0.188	-0.640	-8.360	<0.001
PR	-1.459	0.182	-0.623	-8.021	<0.001

Multivariate Linear regression model: AHI + confounders (age, weight, BMI, smoking, alcohol use, T, and E2).

OSA, obstructive sleep apnea; BMI, body mass index; AHI, apnea hypopnea index; PR, progressive motility; NP, non-progressive; T, testosterone; PRL, prolactin; E2, estradiol.

After adjustment, AHI was significantly and negatively associated with sperm concentration (β = –0.393, p < 0.001), total motility (β = –0.640, p < 0.001), and progressive motility (β = –0.623, p < 0.001). These findings indicate that increasing OSA severity is independently linked to worsening semen quality. No significant association was observed between AHI and semen volume (β = 0.091, p = 0.355).

## Discussion

OSA has been increasingly recognized as a condition with broad systemic impacts, including on male reproductive health. In our study involving 108 patients with OSA and 84 healthy controls, we found that men with OSA exhibited significantly lower sperm concentration, total motility, progressive motility, and testosterone levels. Furthermore, AHI was negatively correlated with key semen parameters, while no significant correlation was observed between AHI and testosterone levels. After adjusting for potential confounders, AHI remained independently associated with semen quality, suggesting a robust relationship between OSA severity and male reproductive impairment.

These findings are consistent with a growing body of literature linking sleep-disordered breathing to testicular dysfunction. A recent multi-arm randomized trial demonstrated that both sleep deprivation and OSA led to marked declines in sperm concentration and motility, as well as reductions in testosterone, indicating a likely causal role for disturbed sleep architecture in reproductive dysfunction (7). Kim et al. reported similar results in a clinical pilot study, identifying widespread sexual and erectile dysfunction along with reduced sperm quality in OSA patients, suggesting the involvement of both hormonal and vascular mechanisms (8). Likewise, animal models of chronic intermittent hypoxia (CIH), a pathophysiological hallmark of OSA, have revealed reductions in sperm motility, structural testicular damage, and altered antioxidant gene expression, mirroring the human phenotype (23). Our findings extend this evidence by demonstrating that even in young, reproductive-aged men, increasing AHI is independently associated with a stepwise decline in sperm parameters, reinforcing the biological plausibility of OSA-related reproductive harm.

The relationship between OSA and testosterone remains complex and potentially influenced by multiple pathways. While some studies have reported a significant inverse association between AHI and testosterone levels (24–26), others—including our own—have not observed such a clear link after adjusting for age and BMI. Potential mechanisms underlying testosterone suppression in OSA include dysregulation of the hypothalamic–pituitary–gonadal (HPG) axis, Leydig cell dysfunction, altered circadian melatonin secretion, adrenal axis dysregulation, and even gut microbiota imbalance. In particular, sleep fragmentation has been shown to blunt nocturnal testosterone peaks, which are closely linked to REM sleep cycles (27). Several clinical studies support an independent relationship between OSA and male hypogonadism. In a cohort of 104 obese men, serum testosterone levels decreased significantly with increasing OSA severity, and AHI was an independent predictor of lower total and free testosterone after adjusting for confounders (28). A recent meta-analysis involving 1,823 men confirmed that serum testosterone was significantly reduced in OSA patients compared to healthy controls, regardless of age or BMI (29). However, obesity remains a major confounder, and future high-quality prospective studies are warranted to disentangle the direct effects of OSA from those of excess adiposity.

The biological mechanisms linking OSA to impaired semen quality are likely multifactorial. Intermittent hypoxia triggers oxidative stress via excess production of reactive oxygen species (ROS), driven by activation of hypoxia-inducible factor-1α (HIF-1α) and downregulation of antioxidant defenses (30, 31). CIH has also been implicated in promoting germ cell apoptosis, inhibition of spermatogonial proliferation, systemic inflammation, and even epigenetic alterations, including histone modification and DNA methylation of non-coding RNAs. Sleep structure disruption further contributes to reproductive dysfunction: reduced proportions of slow-wave sleep have been associated with testicular damage and poor sperm morphology in both human and animal studies (32).

This study has several limitations that should be acknowledged. First, the cross-sectional design precludes any inference of causality between OSA and reproductive dysfunction. Second, the moderate OSA subgroup had a relatively small sample size, which may have limited statistical power in stratified analyses. Third, sleep architecture and hormone secretion rhythms were not assessed in detail, and future studies may benefit from including these parameters through extended PSG monitoring. Fourth, although our main outcomes were analyzed as continuous variables, the absence of predefined diagnostic thresholds prevented the use of receiver operating characteristic (ROC) analysis in this study. Future investigations involving infertile patients or clinical diagnostic endpoints could explore the predictive value of reproductive biomarkers—such as testosterone and motility—through ROC curve analysis to establish clinically meaningful cut-offs. Finally, our findings were limited to young, reproductive-aged men and may not be generalizable to older populations or those with chronic comorbidities.

## Conclusion

In summary, this cross-sectional study demonstrated that patients with OSA had significantly higher body weight and BMI compared to healthy controls. Key semen parameters, including sperm concentration, total motility, and progressive motility, were significantly reduced in the OSA group, along with lower serum testosterone levels. Stratified analysis by OSA severity revealed that increasing AHI was associated with declines in oxygen saturation (MSpO_2_ and LSpO_2_), a higher prevalence of obesity, and progressive deterioration of sperm quality. Multivariable analysis further confirmed an independent, negative association between AHI and semen parameters. These findings suggest that OSA may adversely impact male reproductive function, emphasizing the need for early recognition and management of sleep-disordered breathing in reproductive-aged men.

## Data Availability

The original contributions presented in the study are included in the article/supplementary material. Further inquiries can be directed to the corresponding author.

## References

[1] Basner M Fomberstein KM Razavi FM Banks S William JH Rosa RR. American time use survey: sleep time and its relationship to waking activities. Sleep. (2007) 30:1085–95. doi: 10.1093/sleep/30.9.1085, PMID: 17910380 PMC1978395

[2] Mireku MO Rodriguez A. Sleep duration and waking activities in relation to the national sleep foundation’s recommendations: an analysis of US population sleep patterns from 2015 to 2017. Int J Environ Res Public Health. (2021) 18:6154. doi: 10.3390/ijerph18116154, PMID: 34200277 PMC8201191

[3] Anderson N Tran P. Obstructive sleep apnea. Prim Care. (2025) 52:47–59. doi: 10.1016/j.pop.2024.09.007, PMID: 39939090

[4] Yeghiazarians Y Jneid H Tietjens JR Redline S Brown DL El-Sherif N. Obstructive sleep apnea and cardiovascular disease: A scientific statement from the American heart association. Circulation. (2021) 144:e56–67. doi: 10.1161/CIR.0000000000000988, PMID: 34148375

[5] Benjafield AV Ayas NT Eastwood PR Heinzer R Ip MSM Morrell MJ. Pépin JL et al: Estimation of the global prevalence and burden of obstructive sleep apnoea: a literature-based analysis. Lancet Respir Med. (2019) 7:687–98. doi: 10.1016/S2213-2600(19)30198-5, PMID: 31300334 PMC7007763

[6] Tufik S Santos-Silva R Taddei JA Bittencourt LR. Obstructive sleep apnea syndrome in the Sao Paulo Epidemiologic Sleep Study. Sleep Med. (2010) 11:441–6. doi: 10.1016/j.sleep.2009.10.005, PMID: 20362502

[7] Alvarenga TA Fernandes GL Bittencourt LR Tufik S Andersen ML. The effects of sleep deprivation and obstructive sleep apnea syndrome on male reproductive function: a multi-arm randomised trial. J Sleep Res. (2023) 32:e13664. doi: 10.1111/jsr.13664, PMID: 35670262

[8] Kyrkou K Alevrakis E Baou K Alchanatis M Poulopoulou C Kanopoulos C. Impaired human sexual and erectile function affecting semen quality, in obstructive sleep apnea: A pilot study. J Pers Med. (2022) 12:980. doi: 10.3390/jpm12060980, PMID: 35743765 PMC9225560

[9] Gu Y Wu C Qin F Yuan J. Erectile dysfunction and obstructive sleep apnea: A review. Front Psychiatry. (2022) 13:766639. doi: 10.3389/fpsyt.2022.766639, PMID: 35693968 PMC9178074

[10] Balon R. Burden of sexual dysfunction. J Sex Marital Ther. (2017) 43:49–55. doi: 10.1080/0092623X.2015.1113597, PMID: 26683616

[11] Jankowski JT Seftel AD Strohl KP. Erectile dysfunction and sleep related disorders. J Urol. (2008) 179:837–41. doi: 10.1016/j.juro.2007.10.024, PMID: 18221960

[12] Burschtin O Wang J. Testosterone deficiency and sleep apnea. Sleep Med Clin. (2016) 11:525–9. doi: 10.1016/j.jsmc.2016.08.003, PMID: 28118875

[13] Serrano T Chevrier C Multigner L Cordier S Jégou B. International geographic correlation study of the prevalence of disorders of male reproductive health. Hum Reprod. (2013) 28:1974–86. doi: 10.1093/humrep/det111, PMID: 23670171

[14] Jensen TK Andersson AM Skakkebæk NE Joensen UN Blomberg Jensen M Lassen TH. Rod NH et al: Association of sleep disturbances with reduced semen quality: a cross-sectional study among 953 healthy young Danish men. Am J Epidemiol. (2013) 177:1027–37. doi: 10.1093/aje/kws420, PMID: 23568594

[15] Liu MM Liu L Chen L Yin XJ Liu H Zhang YH. Sleep deprivation and late bedtime impair sperm health through increasing antisperm antibody production: A prospective study of 981 healthy men. Med Sci Monit. (2017) 23:1842–8. doi: 10.12659/MSM.900101, PMID: 28412762 PMC5402839

[16] Wang X Chen Q Zou P Liu T Mo M Yang H. Ling X et al: Sleep duration is associated with sperm chromatin integrity among young men in Chongqing, China. J Sleep Res. (2018) 27:e12615. doi: 10.1111/jsr.12615, PMID: 28994211

[17] Balasubramanian A Kohn TP Santiago JE Sigalos JT Kirby EW Hockenberry MS. Increased risk of hypogonadal symptoms in shift workers with shift work sleep disorder. Urology. (2020) 138:52–9. doi: 10.1016/j.urology.2019.10.040, PMID: 31917971

[18] Levine H Jørgensen N Martino-Andrade A Mendiola J Weksler-Derri D Mindlis I. Temporal trends in sperm count: a systematic review and meta-regression analysis. Hum Reprod Update. (2017) 23:646–59. doi: 10.1093/humupd/dmx022, PMID: 28981654 PMC6455044

[19] Sengupta P Nwagha U Dutta S Krajewska-Kulak E Izuka E. Evidence for decreasing sperm count in African population from 1965 to 2015. Afr Health Sci. (2017) 17:418–27. doi: 10.4314/ahs.v17i2.16, PMID: 29062337 PMC5637027

[20] Wang Z Zhang Q Ding J Yan S Jin W Luo L. Chen H et al: Effect of obstructive sleep apnea on semen quality. Sleep Breath. (2023) 27:2341–9. doi: 10.1007/s11325-023-02847-8, PMID: 37184755

[21] Kandil H Agarwal A Saleh R Boitrelle F Arafa M Vogiatzi P. Editorial commentary on draft of world health organization sixth edition laboratory manual for the examination and processing of human semen. World J Mens Health. (2021) 39:577–80. doi: 10.5534/wjmh.210074, PMID: 34169684 PMC8443989

[22] Sateia MJ. International classification of sleep disorders-third edition: highlights and modifications. Chest. (2014) 146:1387–94. doi: 10.1378/chest.14-0970, PMID: 25367475

[23] Torres M Laguna-Barraza R Dalmases M Calle A Pericuesta E Montserrat JM. Male fertility is reduced by chronic intermittent hypoxia mimicking sleep apnea in mice. Sleep. (2014) 37:1757–65. doi: 10.5665/sleep.4166, PMID: 25364071 PMC4196059

[24] Grunstein RR Handelsman DJ Lawrence SJ Blackwell C Caterson ID Sullivan CE. Neuroendocrine dysfunction in sleep apnea: reversal by continuous positive airways pressure therapy. J Clin Endocrinol Metab. (1989) 68:352–8. doi: 10.1210/jcem-68-2-352, PMID: 2493027

[25] Hammoud AO Walker JM Gibson M Cloward TV Hunt SC Kolotkin RL. Sleep apnea, reproductive hormones and quality of sexual life in severely obese men. Obes (Silver Spring). (2011) 19:1118–23. doi: 10.1038/oby.2010.344, PMID: 21273994 PMC3713783

[26] Gambineri A Pelusi C Pasquali R. Testosterone levels in obese male patients with obstructive sleep apnea syndrome: relation to oxygen desaturation, body weight, fat distribution and the metabolic parameters. J Endocrinol Invest. (2003) 26:493–8. doi: 10.1007/BF03345209, PMID: 12952360

[27] Luboshitzky R Zabari Z Shen-Orr Z Herer P Lavie P. Disruption of the nocturnal testosterone rhythm by sleep fragmentation in normal men. J Clin Endocrinol Metab. (2001) 86:1134–9. doi: 10.1210/jcem.86.3.7296, PMID: 11238497

[28] Tančić-Gajić M Vukčević M Ivović M Marina LV Arizanović Z Soldatović I. Obstructive sleep apnea is associated with low testosterone levels in severely obese men. Front Endocrinol (Lausanne). (2021) 12:622496. doi: 10.3389/fendo.2021.622496, PMID: 34381420 PMC8350060

[29] Su L Meng YH Zhang SZ Cao Y Zhu J Qu H. Association between obstructive sleep apnea and male serum testosterone: A systematic review and meta-analysis. Andrology. (2022) 10:223–31. doi: 10.1111/andr.13111, PMID: 34536053

[30] Li Z Wang S Gong C Hu Y Liu J Wang W. Huang Y et al: Effects of Environmental and Pathological Hypoxia on Male Fertility. Front Cell Dev Biol. (2021) 9:725933. doi: 10.3389/fcell.2021.725933, PMID: 34589489 PMC8473802

[31] Prabhakar NR Peng YJ Nanduri J. Hypoxia-inducible factors and obstructive sleep apnea. J Clin Invest. (2020) 130:5042–51. doi: 10.1172/JCI137560, PMID: 32730232 PMC7524484

[32] Zheng Z Wang H Chen Z Gao H Gao P Gao J. Impact of chronic sleep deprivation on male reproductive health: Insights from testicular and epididymal responses in mice. Andrology. (2024) 13:968-77. doi: 10.1111/andr.13718, PMID: 39092868

